# Partners in Sickness and in Health? Relationship-Centered Veterinary Care and Self-Educated Pet Owners in Germany: A Structural Equation Model

**DOI:** 10.3389/fvets.2020.605631

**Published:** 2021-01-27

**Authors:** Alina M. Küeper, Roswitha Merle

**Affiliations:** Institute for Veterinary Epidemiology and Biostatistics, Freie Universität Berlin, Berlin, Germany

**Keywords:** veterinary medicine, partnership building, relationship-centered care, veterinary- pet owner communication, empathy, shared decision making, structural equation modeling

## Abstract

In recent years, the web has become a widely used source for health information. Pet owners seem to respond to the supply of medical information on the Internet by increasing their self-education. However, after more than a decade of the digital revolution, little is known about the Internet's impact on the veterinarian-pet owner relationship. Recent research has raised concerns regarding the increase in self-education among pet owners. However, reasons suggest that the Internet might be a valuable source of pet-owner education for veterinarians. In particular, relationship-centered approaches of care might benefit from the information provided. Our study aimed to determine the perception of German veterinarians with regard to pet owners' self-education on different aspects of veterinary care. An online survey was conducted for German veterinarians from November 2016 to June 2017. Data were analyzed using exploratory factor analysis and structural equation modeling. Within the structural equation model, we evaluated how the veterinarians' attitude toward relationship-centered care might affect the evaluation of pet owners' self-education. A total of 585 valid questionnaires were completed. The majority of veterinarians (83.6%) welcomed the principles of shared decision-making. Practically, all veterinarians reported a noticeable increase in pet owners' self-education within the last few years. Perceptions on self-education's impacts on veterinary practice varied among the participants. A beneficial impact of self-education was reported regarding the general quality of veterinary care and quality of follow-up care. Most concerns were related to a negative impact on the veterinarian-pet owner relationship and the pet owners' demands on the veterinarians' work after self-education. Moreover, many participants were afraid that unfiltered information may unsettle pet owners and, therefore, advised them against self-education. The structural equation model confirmed the hypothesis that a veterinarian's positive attitude toward shared decision-making, empathic behavior, and his/her evaluation of self-education were associated. Therefore, we concluded that while there are beneficial potentials, there seem to be barriers that prevent the effective use of the Internet as a supportive medium in veterinary care. Further research and training are needed to enable the use of the Internet as an ancillary medium.

## Introduction

Communication and relationship-building are key components of medical encounters (1–3). Therefore, finding ways to improve doctors' communication skills and establish trustful relationships has recently become an important research focus in human and veterinary medicine (4–12).

During the last decades, there has been a fundamental shift within the doctor-patient relationship (13). Instead of a paternalistic, guardian-type relationship, which is described by active and authoritarian physicians and passive patients, great effort has been made to engage in collaborative and relationship-centered philosophies of patient care (14–16). Relationship-centered communication and care improved patient compliance (17), health outcomes (18, 19), and patients' and doctors' satisfaction (17), while the number of malpractice claims reduced (20–22).

Historically, friends and family played a key role during challenging healthcare decision-making; however, nowadays the Internet offers an unlimited source of medical information and supports the patients' quest for autonomy (23–26). It combines the advantages of providing comprehensive and targeted health information in various layperson-friendly formats (videos, infographics, texts, pictures) with a 24/7 availability. Additionally, it offers a low inhibition threshold to ask questions and provides a feeling of privacy (27–29). Evidence supports that self-education provides a chance to improve patients' health literacy and assist patients in making informed decisions about their health. (30) However, there are concerns that self-education can also misinform and unsettle patients, which can lead to mistrust and negatively impact the doctor-patient relationship (24, 25, 31).

Although research on the changes in the veterinarian-pet owner relationship is limited, several studies have shown that pet owners increasingly wish for participation and self-education (32–36). Today, for most pet owners in the western world, the pet plays the role of a friend or family member (37–39). The deeper the emotional bond between the pet and owner, the higher the pet owners' expectations from veterinary health care, regardless of the emerging costs (40, 41). Pet owners' satisfaction with a consultation influences compliance, therapy adherence, and has an important influence on the pets' health (42). Studies indicate that satisfaction can be increased through effective communication, partnership-building (43), and meeting pet owners' expectations (44). Pet owners‘expectations include a respectful and friendly environment, the recognition of their pet's individuality, high quality medical care, and transparency in the communication of relevant information such as treatment options and costs (40, 44–46). Moreover, there should be an opportunity to express sorrow and fears (45). Therefore, a caring and kind veterinarian, respectful treatment, and sharing of information are the most important factors that influence pet owners when choosing a veterinarian (37). A recent study on bird owners in Germany indicated that non-adherence to therapy increased when a bird owner had doubts regarding the diagnosis or therapy, felt uninformed, or did not trust the veterinarian (47).

The implementation of relationship-centered care (RCC) satisfies the specified needs and reflects on the recent veterinary social sciences (42, 48, 49). Shaw described RCC in veterinary medicine as a joint venture between a veterinarian and pet owner with the aim of ensuring the best care for the pet (50). Relationship-centered care consists of four core statements: (1) relationships should incorporate the entire personhood of the participants, (2) emotions are an important component of these relationships, (3) veterinarians and pet owners mutually influence each other, and (4) it is morally valuable to form genuine relationships in health care (51). A “Four Habits Approach” was developed to build a conceptual framework based on the principles of RCC, which can be easily implemented in daily medical practice (2). The first step is to invest in the anamnesis and use open-ended questions and active listening to establish a welcoming atmosphere and elicit all relevant biomedical information regarding the case. In the second step, the veterinarian should understand the pet owners' perspective to develop a mutual partnership, show respect for the counterparts' experiences, and compare similarities and differences in understanding. The third step includes showing empathy, including respect, care, and compassion, as a core conceptual basis of the healing relationship. While these first three steps strive for information gathering, the fourth step supports the sharing of information. Besides delivering relevant diagnostic information, veterinarians are required to involve patients in a participative decision-making process. Finally, the veterinarian should check for the pet owners' agreement and understanding. The goals of this approach are to establish rapport and build trust in a short timeframe, allow an effective exchange of information, and show compassion and concern. This should increase the likelihood of adherence and positive health outcomes (2).

When pet owners have access to valid and understandable information, self-education using the Internet might offer a valuable resource for RCC, especially when the explanation of complex medical contexts would require an excessive amount of time during an appointment. A better understanding of the medical basics might empower pet owners to speak more openly to their veterinarian, ask questions, and make informed decisions (52). However, self-education might also create some bias in the pet owner that influence the decision-making process, especially if wrong information are not addressed during the consultation (53, 54).

Data to describe the use of the Internet for pet health information are available from the United States (US), British, and Australian pet owners. Queried pet owners ranked the Internet as the third most common source of information after veterinarians and veterinary specialists in 2008 and 2012 (55). In 2017, US and British pet owners reported that the Internet was the most frequently used source for pet health information followed by veterinarians (34, 56, 57). In a 2011 study by Bayer, 39% of pet owners reported that they first searched online to check if their pet needed a veterinarian (54). Lofgren et al. reported that 86% of American horse owners utilized the Internet for health information (57). Kogan et al. proposed that pet owners are interested in two main categories of pet health. The first category includes questions regarding “disease and treatment” such as certain medical procedures or alternative medicines and treatments. The second category includes information about health and prevention, such as diets, nutrition, vaccinations, or fitness. When asked for reasons why they access online pet health information, pet owners reported a desire for more information, such as second opinions, clarifications or additional information, interest in pet health in general, or social support from people with similar pet health issues (35, 58). In particular, social media allows an exchange of experiences with “fellow sufferers” irrespective of distances or time zones (59). Another possible motive is that pet owners disagree with their veterinarian and/or do not believe in the information provided (58).

Research on veterinarians' perception of pet owners' use of web-based information sources and how veterinarians estimate the risks and benefits of self-education is limited. In three comparable studies, Kogan et al. asked veterinarians from the US, United Kingdoms, and Australia about their perceptions and experiences related to online pet health information (33, 35, 60). The results show a general division of opinions regarding the impact of online self-education, with some minor differences between the different nationalities. The majority of veterinarians thought that the Internet has a negative or very negative impact on the veterinarian-pet owner relationship, but ~one-third of them described a positive impact. While ~half of the respondents believed that the Internet has a negative impact on pet health (the proportion is considerably smaller in the US), more than one-third found this to be positive. A narrow majority of veterinarians had the impression that they needed to spend more time with “self-educated” pet owners, while the remaining did not perceive any changes in the amount of time (60). Many veterinarians reported a lack of understanding surrounding medical information as a problem associated with self-education of pet owners (33, 35, 60). This could be attributed to a general lack of health literacy in laypersons (30) and the inappropriate use of medical language in the information materials (61, 62). As another problem, veterinarians believed that pet owners might trust inaccurate or misleading information, (33, 35, 55, 56, 63, 64) which will lead to misinformation or result in a belated consultation (33).

One of the foremost expectations that pet owners have from the communication during veterinary appointments is to receive information. This causes a feeling of “being cared for” and helps pet owners cope with anxiety and foster hope owing to the knowledge and social support (46, 65, 66). Although the Internet is the most frequently used source of information, veterinarians are still the most trustworthy source of information and medical advice for pet owners (35). Therefore, there is a chance to enhance the veterinary healthcare quality and positively impact veterinarian-pet owner-relationships by means of a guided encouragement of pet owners' self-education (65, 67, 68). However, Kogan et al. reported that nearly half of the surveyed pet owners stated that no recommendations of reliable websites were made by their veterinarians, although more than 90% would visit veterinarian-recommended sources (34, 35). Recommending accurate online information rather than trying to limit the damage of incorrect information provides an opportunity for veterinarians to actively educate their pet owners and meet their need for information (58).

Although research on the usage and influence of web-based information on human medicine has expanded rapidly, knowledge on how pet owners' self-education might reflect on veterinary medicine remains limited. The aims of this study were to evaluate the attitude of German veterinarians with regard to the impact of pet owners' self-education on daily work and veterinarian-pet owner relationship and to identify factors that influence the assessment of pet owners' self-education. A more positive assessment might encourage veterinarians to use web-based information more frequently to support their information-giving, which might improve pet owners' health literacy and strengthen the veterinarian-pet owner relationship.

## Materials and Methods

### Research Design

A cross-sectional quantitative survey was conducted among German veterinarians. The perception of RCC-related patterns during veterinary appointments and the influence of pet owners' self-education on the veterinarian-pet owner relationship were evaluated. Exploratory factor analysis was conducted to identify latent factors underlying the collected data and to design a preliminary model. In the second step, structural equation modeling (SEM) was performed to confirm the model assumptions (69).

### Questionnaire Design

The questionnaire development incorporated relevant aspects of relationship-centered veterinary care and pet owners' expectations from the literature. Validated questionnaires developed in human medical research were included and linguistically adapted for veterinary medicine (e.g., replace “physician” by “veterinarian”) (31, 70, 71). Items included in the model were measured on a 6-point Likert scale with an additional “no answer” option.

One goal of our study was to evaluate how German veterinarians estimated the impact of self-educated pet owners. From 2003 to 2016, a longitudinal study was conducted on German physicians to assess how they perceived the dynamics of physician-patient relationships under the influence of increasing self-education (31, 72–74). Items to measure veterinarians' perceptions were derived from this questionnaire.

Several relationship-centered models of medical decision-making have been developed in the field of human medical sociology during the last decades (75). The model of shared decision making (SDM) has gained importance in medical care as it improves medical outcomes and patients'/pet owners' and physicians'/veterinarians' satisfaction (76–80). Measurement items to assess SDM were derived from veterinary and human medical research and included in the questionnaire (66, 70–72, 80).

Expressing empathy with the pet owner is a basic requirement to establish RCC during veterinary appointments (51, 81). Qualitative studies on pet owners' expectations suggested that they long for compassion and empathy in communication, respect for their individuality, kindness, and an opportunity to disclose health problems, concerns, and worries (80). Additionally, we expected empathy for the pet to be a relevant factor for pet owners. Accordingly, items that were expected to measure the manifestation of empathy during a consultation were created and added to the questionnaire.

Finally, providing information is one of the foremost requirements that support pet owners in an actively engaging decision-making process. Items measuring veterinarians' motivation to share information were derived from literature and from discussions with veterinarian practitioners while pretesting the questionnaire.

All items used for statistical analysis and the referred literature are summarized in Table 1.

**Table 1:** Questionnaire items (translated to English) and corresponding references for a survey on German veterinarians' perception of aspects of relationship-centered care and the influence of pet owners' self-education.

**Variable code**	**Questionnaire item**	**Reference/adapted from**
V1	Pet owners should be asked whether they want to take part in the medical decision making, before I start diagnostic tests and/or therapeutic measures.	Böcken (31), Stoewen et al. (80)
V2	Pet owners should be encouraged to describe individual characteristics of their pet.	Tresolini (82)
V3	I attach importance to ask the pet owners for a precise anamnesis.	Kurtz and Silverman (83)
V4	I explain the pros and cons to all diagnostic tests to the pet owner.	Scholl et al. (70)
V5	I explain all diagnostic findings to the pet owner.	Scholl et al. (70)
V6	I explain the possible therapeutic measures with the pros and cons for each.	Scholl et al. (70)
V7	I weight the available therapeutic options for each pet together with the pet owner.	Scholl et al. (70)
V8	During the anamnesis I ask pet owners, whether they already informed their self before the consultation.	Kogan et al. (34)
V9	In general, therapeutic decisions should consider the unique situation of pet and pet owner.	Tresolini (82)
V10	Pet owners should be encouraged to express feelings/sorrows during a consultation.	PO like to/should be able to disclose concerns (46, 80, 84, 85)
V11	Pet owners should be asked for permission before manipulating the pet (e.g. make an injection, shearing).	Discussion/Expert Reviews
V12	I try to avoid using medical language while talking to the pet owner.	Coe et al. (46)
V13	The social interaction with pet owners is one of the positive things in my daily practice.	Discussion/Expert Reviews
V14	To be involved into the decision making process is mentally stressful for many pet owners.	Discussion/Expert Reviews
V15	Many pet owners struggle with detailed information about medical issues.	Discussion/Expert Reviews
V16	I advise pet owners against self-education.	Discussion/Expert Reviews
V17	Self-education often unsettles pet owners.	McElroy and Shevlin (86)
V18	I explain to the pet owner which undesirable side-effects may occur during the therapy.	PO like to/should be provided with information. (46, 87)
V19	Before a general anesthetic, I inform pet owners about all possible risks.	PO like to/should be provided with information. (46, 87)
V20	I inform pet owners about the approximate costs that will be caused by the treatment.	Coe et al. (45)
V21	I bill the time needed for counseling interviews in accordance with the German veterinary fee schedules (GOT).	Discussion/Expert Reviews
V24	If I know that pet owners like to inform their selves I try to inform them extra comprehensively.	Böcken (31)
V25	I provide valid, plain information material on common pet healthcare issues for the pet owners.	Stoewen et al. (66)
V26	I recommend my pet owners where to find valid pet health information.	Kogan et al. (34)
V28	In my opinion, a pet owners' interest for pet health-topics is a positive thing.	Discussion/Expert Reviews
V29	Self-education of pet owners has a positive impact on the time needed for a consultation.	Böcken (31)
V30	In general, self-education of pet owners has a positive impact on the quality of pet healthcare.	Böcken (31)
V31	Self-education has a positive impact on the quality of medical aftercare done by the pet owner.	Böcken (31)
V32	The growing amount of available information has a positive impact on the veterinarian-pet owner-relationship.	Böcken (31)
V33	Self-education has a positive impact on the demands/expectations of veterinary care.	Böcken (31)

*PO, pet owner. Gray font: items excluded from the final model*.

Additionally, questions on demographics and years of working experience were added to the questionnaire addressing working conditions (practice/clinic, type of animals treated, number of veterinarians in the team, employee or self-employed). At the end of the questionnaire, participants were able to include individual comments in a voluntary comment field.

The questionnaire was validated using a three-step pretesting process with expert interviews, cognitive pretesting, and standard pretesting. In the first step, the questionnaire draft was discussed with interdisciplinary experts (veterinary practitioners and scientists, psychologists, and social scientists). They were asked to evaluate all the items for relevance within the context of the veterinary-pet owner communication in daily veterinarian practice and with regard to the methodology of SEM. Expert interviews were followed by a cognitive pretest with eight veterinarians. During the cognitive pretest, all items were checked for validity using methods of paraphrasing (repeating the question in own words after reading) and thinking aloud (verbalizing all thoughts and feelings during the answering-process to check out for barriers or misunderstandings) (88). The final quantitative pretest included 22 participants (89).

The final questionnaire comprised 65 items querying general and demographic data.

### Data Collection

A nationwide survey among German veterinary practitioners was conducted from November 14, 2016 to June 30, 2017. Veterinarians who worked in a curative veterinary practice or clinic during the last two years were eligible to participate. Data collection, storage, and processing were completed in accordance with the current German data protection laws. Each participant was adequately informed of the aims, methods, and scope of the survey, and informed consent had to be given actively before the survey could be started. Participation was voluntary. Data collection was anonymous, and no individual-related nor other sensitive data were collected. Participants were free to terminate the survey at any point. Therefore, approval by an ethics committee was not required as per the local legislation.

The questionnaire was distributed online (LimeSurvey, open-source, hosted on university servers) to include a large number of participating veterinarians. A professional information website (www.fokustiergesundheit.de) with an external link to the survey page was developed along with a web designer and was promoted across veterinarians' Facebook groups. Federal Veterinary Associations and the German Association of Practicing Veterinarians (bpt) supported the study by sharing the link in their newsletters and on their websites. A nationwide advert was published in the journal “Deutsches Tierärzteblatt.” Nonetheless, the study population cannot be regarded as representative because of the convenience of the sampling strategy.

### Data Analysis

Data were extracted from the LimeSurvey and the hardcopy questionnaires, stored in Microsoft Excel® version 2016, and statistically analyzed using SAS version 9.4 (SAS Institute Inc., Cary, NC, US).

Items used for modeling were checked visually for normality (histogram, Q-Q-plot) and with Kolmogorov-Smirnov tests. Normality was not established for all items. Descriptive statistics were calculated for all relevant variables, including mean, min, max, standard deviation, kurtosis, and skewness.

Missing data imputation was performed in cases of occasional missing answers in preparation for multivariate data analysis.

#### Exploratory Factor Analysis

In the first step, *N* = 299 observations were chosen randomly and analyzed using exploratory factor analysis (EFA) to characterize the underlying constructs and build a data-driven measurement model. Estimations were done using the proc calis statement with squared multiple correlations as prior communality estimates, followed by a promax (oblique) rotation. As not all the items had a normal distribution, the unweighted least squares method was used.

The Scree test and eigenvalues were used to select the suggested number of factors. Factors with an eigenvalue >1 and before a significant break in the scree plot were kept within the model (90).

#### Structural Equation Model

Based on the results of the EFA, we performed SEM with directional paths between latent factors to confirm the model assumptions.

Latent variables were labeled as F1 (*shared decision-making* factor), F2 (*perceived impact of pet owners' self-education* factor), and F3 (*expression of Empathy* factor). As recommended, each latent factor was measured by at least three indicator variables labeled by the letter “V”; Error terms were named using the letter “E,” and disturbances for endogenous latent factors were labeled by the letter “D” (91).

The model was estimated using the unweighted least-square method in the CALIS procedure (92). Calculations were performed based on the remaining N=262 observations. For each latent construct, the estimate of the factor loading of the variable with the highest loading on this factor in EFA was fixed to 1 in the linear equation (V5 for Factor 1, V30 for Factor 2, and V9 for Factor 3).

Root mean square error of approximation (RMSEA), standardized root mean square residual (SRMR), and Bentler comparative fit index (CFI) were used to assess the goodness-of-fit of the model. For a model fit to be good, the upper level of the confidence interval of RMSEA and the value of SRMR should not exceed 0.05, and CFI should be > 0.91 (69, 93).

Factor loadings, *t*-values, and indicator reliability (*R*^2^) for each variable and the composite reliability for the respective factors were calculated. Variance extracted estimates for each indicator variable were analyzed to measure the amount of variance captured by each construct. Significant *p*-values indicate that factor loadings differed significantly from zero in the large-sample *t*-test (*p* < 0.01). An indicator reliability above 0.39 was considered ideal. Composite reliability analogous to Cronbach's alpha is said to be good if α > 0.70 and ideal if α > 0.80 (69). Variance extracted estimates were considered for each latent construct and for all constructs combined by computing the arithmetic mean and said to be good if in excess of 0.50 (94).

A Lagrange multiplier was used to check whether allowing additional covariances in the model would significantly improve the model fit (69).

## Results

### Sample

In total, 804 questionnaires were returned. Of which, 219 were classified as incomplete (<40% of the items completed) and excluded from the study. The remaining 585 participants had an average age of 42.4 years; 79.5% were females and 20.5% males. Forty-three percent of the participants were employed and 56.9% were self-employed. Approximately 66.7% specialized in the treatment of small animals; 7.3% treated horses; 3.8% treated farm animals; 1.2% treated birds, amphibians, and reptiles; and 21.1% worked in mixed practices. Most of the participants worked in practices with only one veterinarian (30.8%) or two to three veterinarians (37.7%). Seventeen percent worked in practices with more than three veterinarians, and 14.6% worked in a veterinarian clinic. More than two-thirds of the participants had more than 6 years of practical work experience (69.2%), 18.3% had worked for 2 to 6 years, and 4.8% had 1 to 2 years of working experience. Eight percent had <1 year of working experience in a veterinary practice.

### Descriptive Analysis

#### Shared Decision Making

While 86% of veterinarians favored the shared-decision-making model, 10.1% preferred a paternalistic and 3.7% a professional-as-agent model of medical decision-making. Ninety-nine percent of the participants perceived a change in pet owners' information-seeking behavior in the recent years owing to the digital revolution. All the veterinarians reported that in their experience, Internet forums were a common information source for pet owners. Eighty-five percent named friends and acquaintances as common sources and 83.3% perceived information websites to be a source of self-education. Sixty-seven percent named Facebook groups as a perceived information source. Other veterinarians (32.7%), relevant specialized literature (10.1%), and advanced training courses (2.5%) were less common sources of self-education from the veterinarians' view.

#### Dealing With Pet Owners' Need for Information

When asked to describe their information-giving behavior, nearly half (47.7%) of the participants strongly agreed (17.3%) or agreed (30.4%) that they provided plain, valid information materials (e.g., pamphlets) for pet owners, while 22.7% more or less agreed and 16.3% more or less disagreed. More than one-tenth of the veterinarians reported not providing such information to their pet owners (6.8% disagreed, 6.6% strongly disagreed). Furthermore, 41.8% of the veterinarians agreed or strongly agreed when asked whether they shared recommendations with pet owners regarding appropriate information on pet health care and 25.2% more or less agreed. One-third of the participants seemed to not usually give any recommendations of that kind (17.7% more or less disagreed, 9.3% disagreed, and 6.0% strongly disagreed).

**Figure 1:**
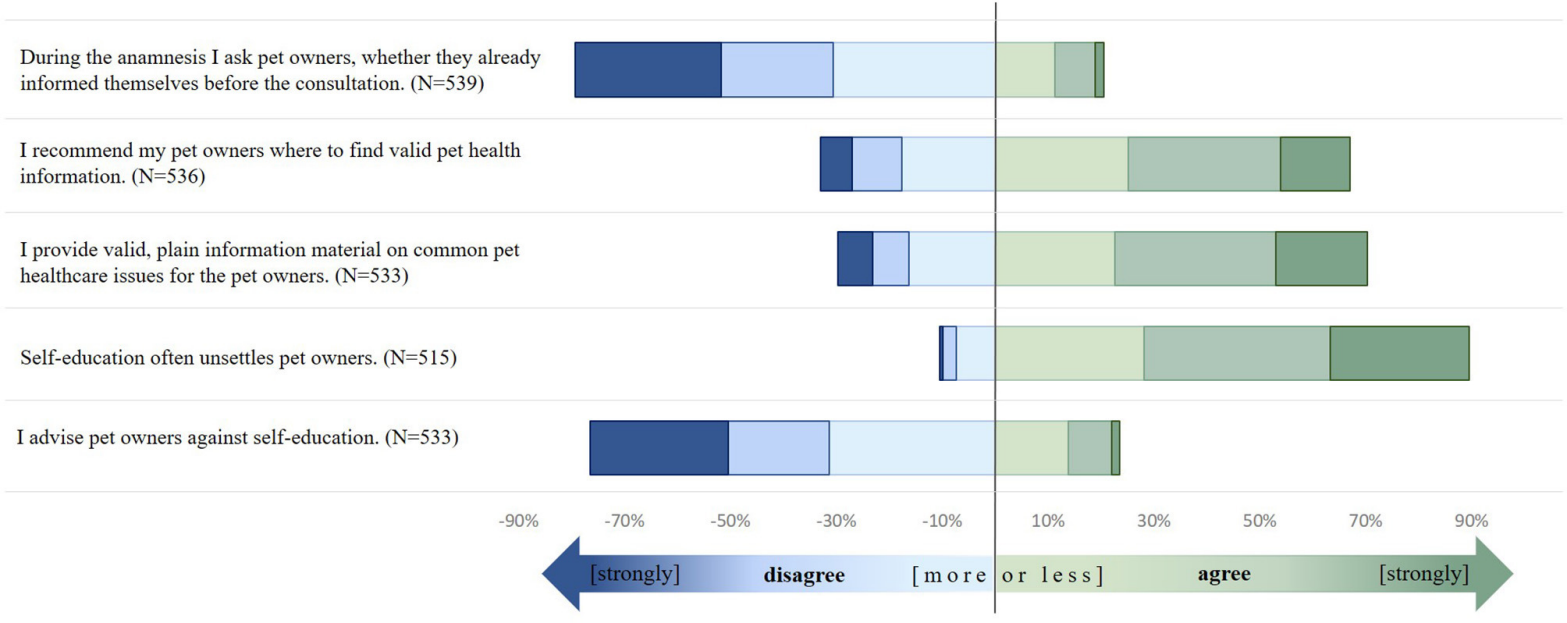
German veterinarians' information giving and recommendations during veterinary appointments. Survey on German veterinarians (2016–11/2017–06). Answers had to be given on a likert scale (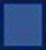
1 = strongly disagree, 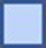
2 = disagree, 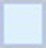
3 = more or less disagree, 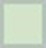
4 = more less agree, 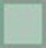
5 = agree, 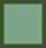
6 = strongly agree).

More than one quarter of the participants (26.2%) strongly agreed that self-education often leads to uncertainties among pet owners. Additionally, more than one-third (35.4%) agreed with this statement and nearly another third more or less agreed (28.1%). Twenty-three percent tended to advise their pet owners against self-education (9.6% strongly agreed/agreed, 13.7% more or less agreed). Seventy-nine percent of the veterinarians reported that, more or less, they did not evaluate during the anamnesis whether a pet owner had already informed him/herself. The results are displayed in Figure 1.

#### Perceived Impact of Self-Information

Nearly 80% of the respondents described pet owners' interest in pet health and their need for information as “very positive” or “positive.” Another 14.5% described it as “more or less positive.” Approximately 70% of the responding veterinarians believed self-education is beneficial for the quality of pet health care in general and the quality of the pets' aftercare/therapy done by the owners.

However, the majority of veterinarians noted that pet owners' self-education influenced the time that is needed for explanations with 41.6% perceiving “more or less negative” influence, 8.7% “negative” (8.7%), and 1.5% “strongly negative,” while 22.0% had the impression that significantly less time was needed to explain things if pet owners' informed themselves (“very positive” or “positive” influence). Nearly half of the respondents perceived a “more or less negative” impact of pet owners' self-education on the demands/expectations they had of the veterinarian's work. Another 16.6% perceived this impact as “negative” or even “highly negative”.

**Figure 2:**
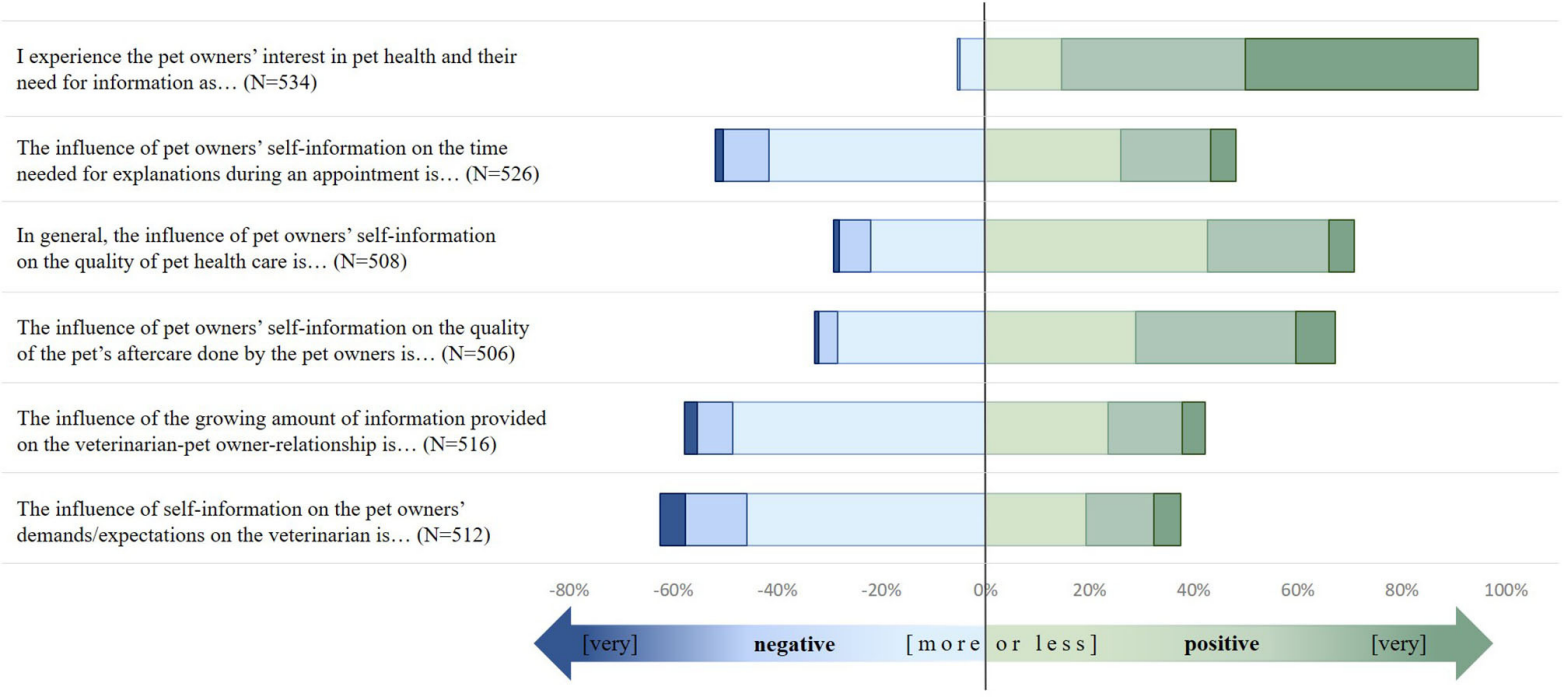
German veterinarians' perception of the impact of pet owner's self-information on different aspects of veterinary care. Survey on German veterinarians (2016–11/2017–06). Answers had to be given on a likert scale (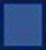
1 = strongly disagree, 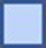
2 = disagree, 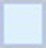
3 = more or less disagree, 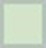
4 = more less agree, 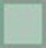
5 = agree, 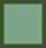
6 = strongly agree).

More than half of the veterinarians reported concerns surrounding pet owners' self-education. Of those, nearly one-tenth estimated the self-information's impact to be “negative” or “highly negative” On the other hand, 18.6% of the respondents perceived self-information as a benefit for strengthening the relationship with pet owners. The results are displayed in Figure 2.

### Exploratory Factor Model

The scree test and eigenvalues suggested three latent factors. In interpreting the rotated factor pattern, an item was defined as loading on a given factor if the factor loading was at least 0.45 for that factor and <0.45 for another.

**Figure 3:**
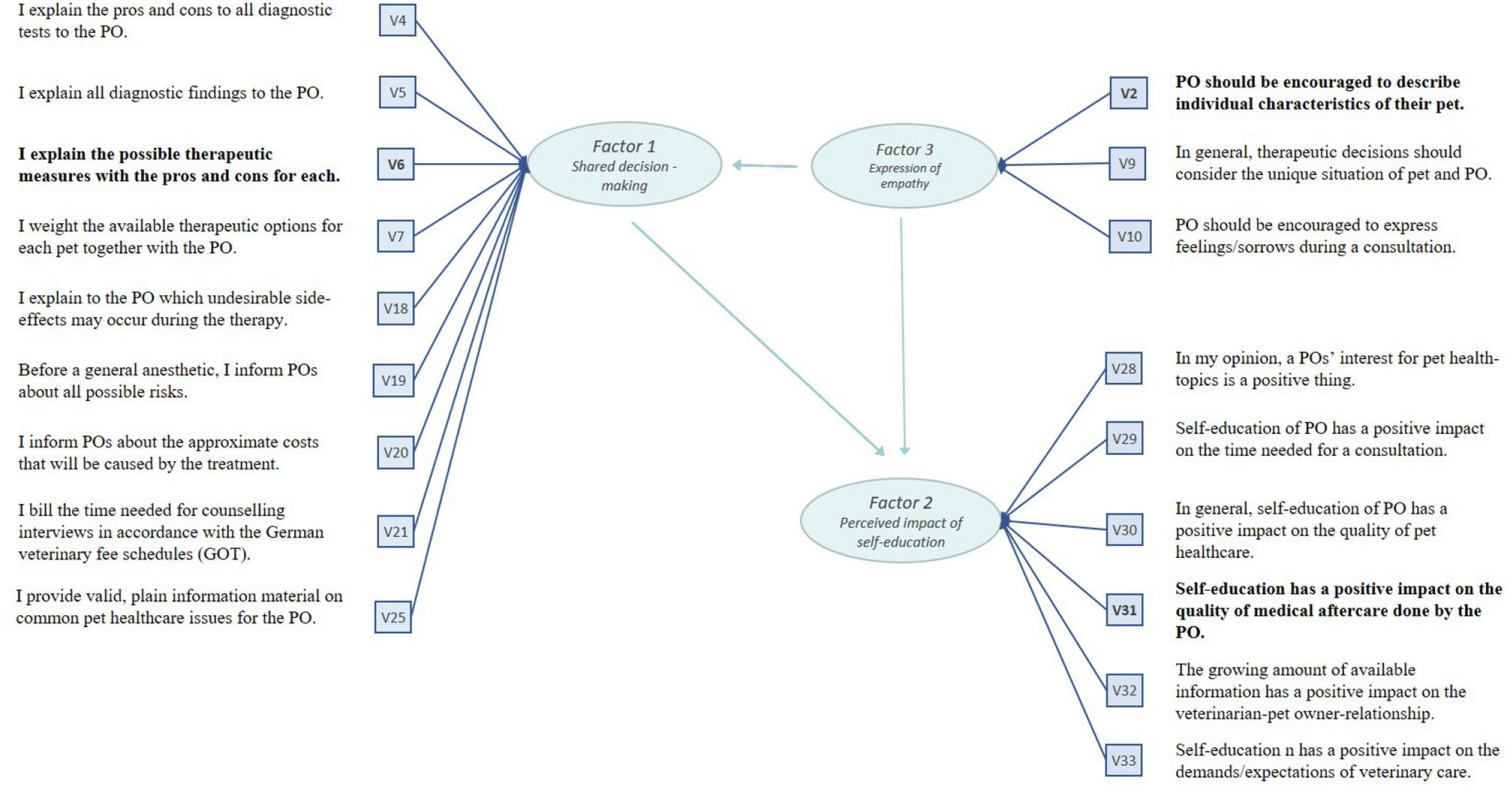
Measurement model to evaluate associations between German veterinarian's information preferences and their perceived impact of pet owner's self-education. Model assumptions based on results of Exploratory Factor Analysis. Factor labels reflect the hypothesized underlying latent construct measured by the questionnaire items. PO = power owner; V = measurement item; bold font = measurement items with highest value of standardized regression coefficient. Calculation dine with SAS version 9.4, *N* = 262.

Applying these criteria, 15 items (V1, V3, V8, V11, V12, V13, V14, V15, V16, V17, V22, V23, V24, V26, V27) were no longer considered. Values of the standardized regression patterns are presented in Table 2. The factors were named in accordance with the subject of the respective measurement items. Nine items were found to load on the first extracted factor. All items measured different aspects of the SDM process; therefore, the factor was named *the Shared decision-making* factor (F1). Six items were found to load on the second factor, which was named *the perceived impact of pet owners' self-education* factor (F2). The remaining three items loaded on the third factor, which was labeled *as the expression of empathy* factor (F3). The model is shown in Figure 3.

**Table 2:** Standardized regression factors.

	**Factor 1**	**Factor 2**	**Factor 3**
**Standardized factor pattern (Standardized regression factors)**			
V1	5	0	28
V2	−9	14	65*
V3	39	1	3
V4	58*	−6	13
V5	59*	−4	10
V6	54*	−5	22
V7	46*	1	33
V8	34	12	−12
V9	−2	−4	67*
V10	3	3	62*
V11	34	11	16
V12	29	4	9
V13	29	22	10
V14	−20	−11	28
V15	−34	−21	29
V16	18	−28	2
V17	1	−3	16
V18	57*	−6	16
V19	58*	−10	−6
V20	54*	−4	−5
V21	52*	−2	−8
V22	31	−7	7
V23	31	1	17
V24	11	−12	25
V25	53*	0	−21
V26	44	13	−22
V27	31	13	−2
V28	9	49*	−4
V29	3	65*	2
V30	−9	78*	−5
V31	−2	76*	−4
V32	6	70*	13
V33	6	62*	15

Results of an Exploratory factor analysis to analyze a survey on German veterinarians' perception of pet owners' self- education. Estimations were done with SAS vs. 9.4, using the proc calis statement with squared multiple correlations as prior communality estimates, followed by a promax (oblique) rotation. Unweighted least squares method was used. N = 233. Values multiplied with 100; values > 0.45 are marked with “*”.

### Structural Equation Model

For each latent construct, the variable with the highest loading on this factor in EFA was selected to be fixed to 1 in the linear equation (V6 for Factor 1, V31 for Factor 2, V2 for Factor 3).

Standardized factor loadings, *t*-values, and indicator reliability (*R*^2^) for each variable and the composite reliability for the respective factor are presented in Table 3. Standardized loadings ranged from 0.14 (V21) to 0.81 (V31), with all being statistically significant at *p* < 0.01.

**Table 3:** Properties of the final model.

**Latent constructs and Indicators**	**Standardized loading**	***t***	**Reliability**	**Variance extracted estimate**	**Error variance (1-R2)**
*Shared decision making [F1]*			0.76^b^	0.30	
V4	0.72	29.16	0.52		0.48
V5	0.56	18.26	0.31		0.69
V6	0.80	38.40	0.64		0.36
V7	0.76	31.38	0.58		0.42
V18	0.69	23.13	0.48		0.52
V19	0.19	2.94	0.04		0.96
V20	0.35	5.96	0.12		0.88
V21	0.20	3.09	0.04		0.96
V25	0.14	2.07	0.02		0.98
*Perceived impact of pet owners' self-education [F2]*			0.82^b^	0.48	
V28	0.48	13.34	0.23		0.77
V29	0.66	24.06	0.44		0.56
V30	0.78	36.43	0.61		0.39
V31	0.81	40.03	0.66		0.34
V32	0.66	24.06	0.44		0.56
V33	0.55	16.86	0.30		0.70
*Expression of empathy [F3]*			0.63^b^	0.37	
V2	0.71	12.73	0.50		0.50
V10	0.40	26.69	0.16		0.84
V14	0.67	11.89	0.45		0.55

*Latent constructs and indicator variables of a structural equation model measuring the relations between aspects of relationship-centered care and German veterinarians' perception of pet owners' self-information*.

b *= Composite reliability of the factor (α)*.

Goodness-of-fit indices showed improved model fit (RMSEA = 0.067 (confidence interval 0.061 – 0.072), SRMR = 0.072, and CFI = 0.83).

Indicator reliability was ideal (>0.39) for items V2, V4, V6, V7, V10, V18, and V29 – V32. Values for items V5, V9, V14, V19, V20, V21, V25, V28, and V33 were notably improved. Composite reliability (marked with ^b^) was good for Factor 1 (α = 0.76), excellent for Factor 2 (α = 0.82), but should be regarded critically for Factor 3 (α = 0.63).

**Figure 4:**
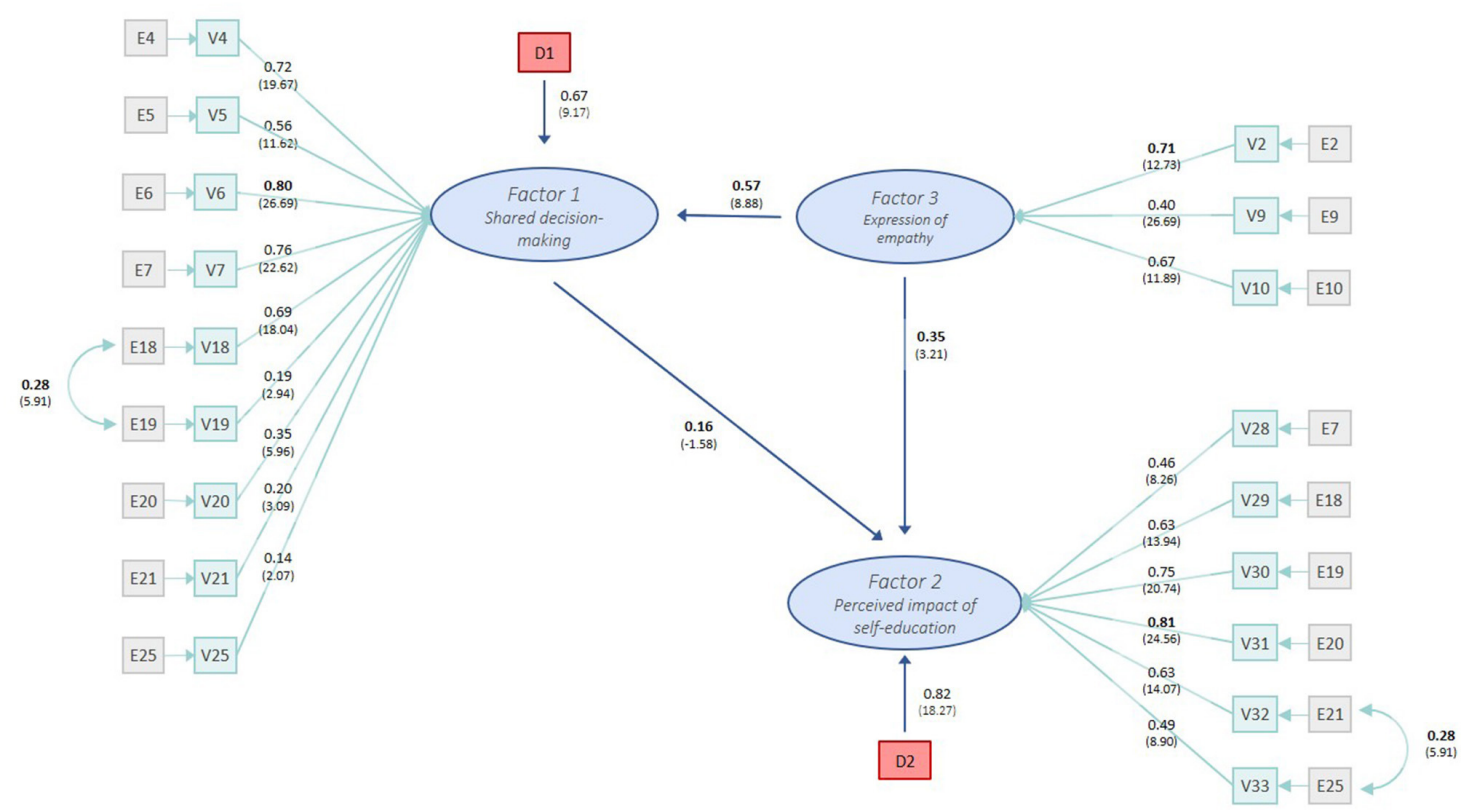
Structural equation model with standardized factor loadings and *t*-values in brackets. Survey on German veterinarians' perception of the actual communication with self-educated pet owners. F, latent factor; V, measurement item; E, error; D, disturbance. *N* = 262.

The Lagrange multiplier suggested that the covariance between E18 and E19 and between E32 and E33 significantly improved the model fit. This adaptation could be justified on a theoretical basis and, therefore, was implemented. Goodness-of-fit indices of the revised model showed an acceptable model fit with RMSEA slightly higher than 0.05 (0.051 confidence interval 0.046 – 0.056), SRMR <0.08 (0.066), and CFI > 0.91 (0.912). Variance extracted estimates were improvable for all factors with an acceptable overall value of 0.39 for all constructs combined. As no more reasonable changes could be done, the model was retained for discussion, as shown in Figure 4.

#### Shared Decision-Making Factor (F1)

The *shared decision-making* factor comprised items derived from a validated tool to measure SDM in human medicine and the Calgary Cambridge Guide for Medical Interviews (70, 71). The factor was mostly associated with the veterinarian's motivation to explain the given therapeutic options, including their pros and cons (V6), followed by the practice of making a joint consideration of all the therapeutic options with the pet owner (V7). Explanations of the pros and cons of further diagnostic tests (V4), the therapy's side effects (V18), and the diagnostic findings (V5) showed high associations with the *shared decision-making* factor. Billing counseling interviews in accordance with the veterinary fee schedules (GOT) (V21) and providing valid, plain information material on common pet healthcare issues (V25) showed less high but still significant associations with F1.

#### Perceived Impact of Pet Owners' Self-Education Factor (F2)

The six items that loaded on the *perceived impact of pet owners' self-education* factor were derived from a German longitudinal study on how physicians assessed the impact of patients' self-education on medical care (31). The factor showed a highly positive association with the veterinarians' conviction that self-education positively influenced the quality of medical aftercare/therapy performed by the pet owner (V31) and the quality of veterinary care in general (V30). The opinions that self-education decreased the time required for explanations (V29) and had a positive influence on the veterinarian-pet owner-relationship (V32) also showed high positive associations with this factor. The perception that pet owners' self-education might have a positive impact on the demands or expectations of veterinarians (V33) and a positive opinion of pet owners' interest in their pets' health care (V28) showed lower but still significant positive associations with F2.

#### Expression of Empathy Factor (F3)

The *expression of empathy* factor had its highest association with showing respect for the pet's individuality when asking the pet owner to describe the pet's special characteristics (V2). Furthermore, the veterinarian's view that pet owners should get the opportunity to express their feelings during a consultation (V10) and the awareness that therapeutic decisions should consider the unique situation of pet and pet owners (such as feeding routines, working hours, age; V9) were highly associated with an empathic approach.

#### Overall Model

Standardized results for covariance among factors F1 and F3 showed a high correlation between a veterinarian's *expression of empathy* factor and the scope for efforts to implement shared decision-making (*shared decision-making* factor) into daily practice (0.57, *t* = 8.88, *p* < 0.001). The standardized path coefficients connecting the *expression of empathy* factor with the *perceived impact of self-information* factor showed a significant positive factor loading with PF2F3 = 0.35 (*t* = 3.21, *p* < 0.01). The standardized path coefficient for *shared decision making* factor with the *perceived impact of self-education* factor showed a coefficient of 0.16 (*t* = 1.58, not significant).

Consideration of the standardized results for variances of exogenous variables showed that 18% of the variance in the veterinarian's *motivation for information-giving* factor (F2) could be accounted for by the factors F1 and F3 (exogenous disturbance term D3 = 0.82 with *t* = 18.27). Thirty-three percent of the variance in the *shared decision making* factor (F1) could be accounted for by the *expression of empathy* factor (exogenous disturbance term D1 = 0.67 with *t* = 9.17).

## Discussion

Nothing is as constant as change. With the digital development that culminated with web 4.0, the Internet has evolved to be an important source of health information (95). Pet owners seem to respond to the burgeoning supply of information with gratitude and a growing motivation for self-education with regard to their pet's medical issues (34, 56, 58, 96). However, after more than a decade of digital revolution, little is known about the Internet's impact on veterinarians' practice and the veterinarian-pet owner relationship. This study was conducted to capture the perception of German veterinarians with regard to different aspects of self-educated pet owners. In the authors' perception, a veterinarian's negative attitude toward self-educated pet owners might become barriers to effective veterinarian-pet owner interactions. The positive effects of self-education, such as a deeper understanding of their pets' health issues, may be undermined. Therefore, as a primary objective, factors that may lead to a more positive assessment of pet owners' self-education should be identified. To achieve this goal, we investigated associations between positive vs. negative perceptions and acceptance vs. refusal of the relationship-centered veterinary care (sharing decisions, offering emotional support, and appreciation-giving).

Results showed that 98.7% of the participating veterinarians reported an increase in pet owners' self-education behavior within the last few years. Therefore, the increase in self-education is not a silent development and seems to noticeably affect the interaction with the pet owner during veterinary appointments. Based on recent studies, this development has continued for nearly one decade. Studies on British, American, and Australian veterinarians described the upcoming generation of self-educated pet owners (33–35, 97, 98). Since the beginning of the “self-educated pet owner” phenomenon, there have been no fundamental changes in the pet owners' self-education habits and as the veterinarians' perception of its impact on the veterinarian-pet owner relationship and their daily work (34, 35, 56).

The veterinarians' complains and reported risks remained similar over the years; therefore, no far-reaching solution seems to be available till date. Similar to US and British veterinarians concerns (35, 60), our results indicate that German veterinarians are worried that self-education on pet health issues often unsettles pet owners. More than two-thirds of our respondents “more or less agreed” to this statement. In previous studies, veterinarians raised concerns regarding the Internet as a source for self-education. They were concerned that many pet owners do not fully understand what they read online and might get upset or scared (33, 35). These concerns are supported by research on “Dr. Google's” impact on the mental well-being of patients. Research suggests that self-education might cause serious psychopathologies (“cyberchondria”) when not combined with professional guidance and an adequate level of health literacy to critically assess a source's reliability (86). This is contrary to the results of a study involving US pet owners, where most of the participants reported that their self-education helped them to communicate better with their veterinarian, have a better understanding of their pets' health issues, and make better choices about their pets' health. Pet owners reported feelings of relief, reassurance, and confidence, while feelings of frustration or anxiety were rarely reported (35, 55).

To conclude that self-education should be rejected categorically would be rather shortsighted because it ignores the beneficial potential of guided self-education of pet owners. Guided self-education implies that pet owners should not be left alone with the ideas and questions that come up during their research but should be guided through their information-seeking process. In the case of self-education on the Internet, a human medical study found that patients who had the chance to discuss their online findings with their doctors reported to be more satisfied than patients who did not and reported a positive impact on their relationship with their doctor (25, 99). Surveys on pet owners showed that only a minority had the opportunity to discuss their online findings with their veterinarians (33, 35). As reasons for non-discussion, patients reported that they did not want to challenge the doctor, thought of the information as supplementary only, or found the information confusing or untrustworthy (100). Moreover, doctors reported that it was difficult for them to discuss online findings as they feared that patients might not be open to options other than the ones they read online and they sometimes felt threatened or challenged in their authority (99, 101). In our study, only a minority of veterinarians often inquired about the clients' online research during the consultation. This could further explain why some pet owners seem to avoid discussing online findings during appointments; they might feel that their self-education is not welcome if they are not invited by their veterinarians to openly discuss them. Undoubtedly, it seems more desirable to force open discussions on the ideas and concerns that the pet owners may have after self-education instead of risking uncertainties, mistrust, or a discontinuation of the pet's therapy due to external influences. If pet owners are already embedded in the process of information finding and have developed concrete opinions or are under the influence of primarily belief-based ideas (e.g., homeopathy and bioresonance), it may be quite challenging to counteract them (53). For the sake of effective therapy, it is particularly necessary to aim for a pet owner's compliance in such cases. An adequate way to explore and counter those ideas or fears is to force an open and constructive discussion during appointments. Nevertheless, there seem to be barriers for such open-minded discussions that should be further investigated in interviews or surveys on pet owners and veterinarians.

Clearly, the Internet offers risks that must be kept in mind while working with self-educated pet owners. Currently, most pet owners report almost exclusively the use of search engines for research on pet health (32, 35). This entails a strong information bias owing to website ranking, which is primarily influenced by online marketing measures instead of substantive assessments. Consequently, heavily advertised information websites will be used more frequently than non-advertised websites, which may offer less commercial but more objective information (53). Allam et al. showed in a quite disturbing way how the selection and ranking of search engines subtly affects patients' evaluation of health issues beneficially or detrimentally; they were able to manipulate the participants' knowledge and assessment of vaccination due to differently arranged search results (53). This demonstrates how unguided self-education using the Internet might easily shape wrong expectations and exaggerate fears and underlines the importance of providing clear recommendations for websites with validated contents. Similar influences might be expected from other common information sources such as friends, trainers, breeders. As their information is probably belief- and experience-based, they might interfere with evidence-based decision-making. Further research on the influence of sources like personal advice on veterinarian-pet owner relationships might help to draw an accurate picture of how different information sources might affect the interaction during appointments.

It may be hypothesized that fulfilling the pet owners' need for information by providing high-quality plain information material and/or recommending a range of information websites, at least for common diseases, will decrease the riskier self-reliant information-seeking behavior (32). Reciprocal beneficial effects might be expected when pet owners acquire the same information verbally provided by the vet repetitively and consistently from different sources, as long as they do not significantly contradict the pet owners' beliefs and experiences (102, 103). Scientific proof should be supplied for the potential improvement of pet owners' compliance and a decrease of unnecessary uncertainties due to unreliable information.

Fortunately, the results of our study indicate that more than two-thirds of the participants provided valid, plain information material for pet owners and gave recommendations where to find further valid information at least sometimes. Kogan et al. noticed that although most of the queried veterinarians seem to realize that their pet owners' do online research, neither do they foster discussions about results or questions nor do they commonly give any recommendations on where to find valid information (35). However, the majority of pet owners reported that they would likely or very likely follow website recommendations or take advice on how to search the Internet for pet health information (35). Veterinarians confirmed this statement, with 85% perceiving that pet owners likely or very likely would follow-up on their suggestion (35). In our study, at least one tenth of the participating veterinarians agreed or strongly agreed that they explicitly advise pet owners against self-education. For an interested and a motivated pet owner, this might induce a feeling of information being dumbed down, cause frustration, and in the worst-case lead to mistrust. Further research on pet owners' perceptions seems valuable to obtain a better understanding of how fostering respect and suppressing self-education impacts the relationship of trust between pet owners and veterinarians. Although the Internet is the most frequently accessed source for pet owners, veterinarians are still the most trusted source of information (35, 57). Similar results can be derived from human medical research. (104). This leap of faith that is given to the veterinarian in the first place should be actively used to deal with pet owners' motivation for self-education in a constructive way. This should include recommending reliable sources and offering an open-minded discussion of uncertainties and new findings that pet owners might make during their research. This would give the opportunity to foster an environment of trust, mutual respect, understanding and a chance to acquire unknown knowledge; in particular, highly motivated and adequately reflective pet owners with a good health literacy could perform extensive research and contribute to the veterinarian's work.

The remarkably high proportion of veterinarians who perceived the self-education's influence on the time needed for explanations as more or less positive, positive, or very positive (nearly half of the participants) might be due to the abovementioned reason. Overall, the participants of our study evaluated the time factor of self-education more positively than that of comparable populations from prior studies. In a study by Kogan et al., none of the veterinarians reported that self-education using the Internet decreased the amount of time they spent with pet owners but nearly 40% reported an increase in the amount of time needed (35). Unfortunately, in both cases, there is no information about how much time the veterinarians spent per consultation before this phenomenon; therefore, a final assessment of this fact should be done cautiously. Spending more time with each pet owner might be problematic if there is a time crunch during the surgery, but it might improve health outcomes as longer consultation times are associated with higher compliance and adherence to therapies (105).

Opinions on the impact of Internet self-education on the veterinarian-pet owner relationship were divided in previous studies. More than half of the Australian veterinarians perceived a negative impact (56.5% negative, 33% positive) (33), and British veterinarians gave a slightly more positive assessment but still reported more negative (54%) than positive (37%) experiences (60). Only US veterinarians reported a mainly positive perception of the Internet's impact (45.3% positive vs. 32.5% negative) (35). Within our study, 9.1% of the veterinarians reported a negative or very negative influence of the growing amount of information on the veterinarian-pet owner relationship, while another 48.6% perceived it as more or less negative. However, 23.6% perceived a more or less positive and 19.3% a positive or very positive effect.

The impact of discussing findings of self-education on the veterinarian-pet owner relationship strongly depends on how both parties react to their questions and concerns. For the veterinarian, recommending reliable sources and discussing unreliable or disturbing information offers a chance to educate and increase pet owners' satisfaction (55, 102, 106). In fact, veterinarians may benefit from encouraging their pet owners to talk about the information they find online or receive from others and take this as a chance to build a better relationship instead of feeling threatened in their authority. As the Internet will not become less important in the future, the number of pet owners who will research online will not decrease. It is the veterinarians' task to ensure that they take navigate the process and minimize the risks by taking an active role in recommending valuable information sources and making self-education something positive for all parties involved.

Nearly all respondents stated that pet owners' interest in pet health and their need for information is something positive. More than two-thirds assessed self-education to be beneficial for the quality of pet health care in general and the quality of the pets' aftercare/therapy done by the owners. A positive impact of the Internet on the pets' health in general was also reported by US veterinarians; 55.6% of the respondents perceived the benefits, while 23.1% felt a negative impact (35). However, a 2011 study drew a more negative picture as it showed that 15% of pet owners who used the Internet reported that they relied less on their veterinarians and that 66% of veterinarians agreed with the statement that due to the Internet sick or injured pets are often brought in 2 or 3 days later than they used to be (54). This is contrary to human medical studies, which showed some evidence that self-education from the Internet prompted people to contact their healthcare providers (67, 68) and that Internet usage increased the frequency of health professional contacts (103). Lee concluded that Internet research raised the patients' need to seek professional to understand the information; therefore, the Internet can complement professional care, rather than usurp it. (103). Further research is needed to assess the influence of self-education on pet owners' willingness to consult a veterinarian.

The kind of positive influence of self-education on pet health care can only be hypothesized at this point and should be further investigated through qualitative research.

There seem to be widespread concerns that self-education negatively influences pet owners' expectations of veterinarians. Personal and first-hand experiences show that it is quite challenging to provide plain information in a small amount of time. The pet owners' wish for veterinary medical care that is both of high quality and affordable makes time a scarce resource. However, in the long run, teaching pet owners and helping them find valid information on their own might be a way to reduce the time needed for explanations. Another point of conflict might be that it becomes increasingly challenging to keep up with the speed of information spreading. Being updated about all medical questions has gained importance because veterinary medical work becomes verifiable owing to the nearly limitless access to medical information. Again, a culture of open discussion would offer the opportunity to deal with these changes in a constructive way. Further, development might force an increase in professional specialization as seen in the human medical field in recent decades.

Regardless of specialization, caring and compassion form the core conceptual basis of a healing relationship. Empathy is the core skill for enacting it and builds the heart of RCC (1). As the results of our SEM showed, empathy for the pet owners' situation is closely linked with participative decision making and may help to deal with the positive aspects and problems and risks of self-education. The dependency of the factors has been underlined in previous research that highlighted relationship-building as vital to the success of every appointment and found empathy to be a central key for building good relationships (107). To put oneself in the position of the pet owner helps one understand the needs, such as anxiousness, uncertainty, helplessness, or simply a high interest in the pet's health issues, influencing his/her information-seeking behavior. Therefore, empathic behavior opens up ways to fulfill those needs through a trustful and open cooperation between pet owners and veterinarians. False information might be corrected, valuable information might be found, the relationship, the pet owners' health literacy, and compliance might be improved. The pet owners' need to look for alternative information sources might decrease and their faith in conventional veterinary medicine might be empowered (32). Moreover, learning to respect the human needs of pet owners and to be more than “only” a scientist or detective looking for medical solutions but rather a teacher and someone who is trusted and revered for his/her humanity might sustain veterinarians during their emotionally demanding work (76, 108). Nevertheless, recent studies indicate that veterinarians use empathic statements only sporadically during appointments. In a study on veterinarian-pet owner communication, Shaw et al. observed that gathering biomedical information using closed-ended questions dominated veterinarian communication, while empathetic (expressed in 7% of the appointments) and partnership statements (expressed in 2% of the appointments) were underrepresented (5).

Veterinarians might wonder how showing empathy during appointments is possible in the time-limited and sometimes stressful environment of daily practice. It is questionable and worth discussing why veterinarians seem to be forced to work under conditions that stifle empathy and leave no time to invest in partnership-building measures. Shaw et al. measured the mean duration of appointments and found that appointments with primarily relationship-building communication patterns were significantly shorter than appointments with biomedical communication patterns (109). Studies on “outstanding clinicians” showed that they invariably found a way to effectively use “windows of opportunity” to respond to patient emotion (110). Eye contact, a warm tone, a welcoming body posture, and facial expression are effective instruments of empathy that do not require additional time. Moreover, investing in an empathic behavior is valuable for the mental well-being of veterinarians and pet owners (80, 108) and for the reputation and economic success of a practice; empathic behavior results in more satisfied pet owners and higher adherence to therapy recommendations (42, 66, 80) and reduces the need to consult alternative health providers (32). In a study, scientists were able to distinguish physicians who had never been sued and physicians who had been sued for malpractice at least twice by analyzing their tone of speech (21).

Cole and Bird identified five types of verbal statements that convey empathy, which can be used to educate oneself on empathy (111). One can easily train using the suggested generic format. The first type is “Reflection” and requires reflecting on a hint or a “potential empathic opportunity (PEO)” (3) that the pet owner might have given (e.g., “It sounds like you are afraid that…”). The second is “Legitimation” (e.g., “Most of the pet owners struggle with…”), the third is “Support” (e.g., “I will support your decision…”), the fourth is “Partnership” (e.g., “Together we will figure out what is best for…”), and the last is “Respect” (e.g., “I have confidence that you will find the right way for…”) (111).

Concerning the main question of this paper, the results suggest that veterinarians who have a positive attitude toward SDM, especially to empathic behavior, seem more likely to assess the impact of self-education on veterinary care as something beneficial. If veterinarians accept the idea of web-based information sources to be a potentially valuable tool and take advantage of its possibilities, educational projects to promote RCC in veterinary schools might be a valuable approach.

## Conclusion and Outlook

### Limitations

Generalizations of the study results should be done with caution. The study sample cannot be considered representative. During the data sampling, no explicit measures were taken to ensure that all types of veterinarians were evenly represented. As the sampling strategy (online questionnaire) was focused on Internet users, an overrepresentation of veterinarians with a high affinity for web-based information sources is probable. Moreover, due to the regional limitation, valid conclusions can only be drawn for German veterinarians. Nonetheless, the hypotheses might be valid for other countries with a comparable structure of veterinary medical care and dynamics of self-education.

Although the study population was quite large, exploratory factor analysis and SEM would have benefited from a larger sample size. Future study designs should include larger sample sizes to validate the model fit and the results.

Within the model, a large part of the *shared decision-making* and *perceived impact of pet owners' self-education* factors' variances remained unaccounted for (D3 = 0.67, D2 = 0.82). Therefore, it can be hypothesized that there may be some influencing latent factors, which have not been taken into consideration. Further, investigations should reconsider the corresponding extended models. Moreover, the reliability of the measurement constructs must be improved in parts. Items with factor loadings <0.3 should be reconsidered. More appropriate measurement items for each latent variable might be evaluated through qualitative interviews, using a Delphi method design, or a mixed methods approach. Therefore, more relevant measurement items that were not considered sufficiently within this survey could be identified. In combination with a representative sample, more concrete approaches and general statements to foster positive dealing with the web-based self-education could be derived.

Like all quantitative approaches, this study had the ability to miss interesting facets. Thus, the authors suggest evaluating and validating appropriate items for additional factors by means of qualitative interviews with pet owners. Re-evaluation of the items comprising the factors F3 and F4 appears appropriate because its factor loadings and reliability are smaller than that in the other latent constructs and their indicator variables.

### Implications for Further Research and Practical Application

The year this study was conducted (2017) marked a milestone in the digital world; nearly half of the world's population had access to the web and 37% of the global population was networking with each other via social media (112). To take full advantage of the upcoming e-health era, research on the dynamics of the veterinarian-pet owner relationship under the influence of digital information sources seems urgent to actively shape future interactions and drive innovation. The necessities and potentials of a successful “change management” have preoccupied economic science since more than one decade but is not sufficiently reflected in veterinary medicine. The successful implementation of change is closely linked to the need to promote cultural change within enterprises and professions. Therefore, establishing and fostering a culture of trust, focusing on the “human success factor” (employees, “customers”) and driving digital change, and implementing technological potentials turned out to be keys to success within enterprises (113). For veterinary practices, it is necessary to welcome the changing demands of self-informed pet owners. This enables veterinarians to take advantage of the “human factors” instead of disregarding the pet owners' needs (which then might be fulfilled by non-medical health service providers) and risking further undermining of the pet owner-veterinarian relationship. The principles of RCC and a relational coordination seem to offer a promising approach to implement the social factors of success in veterinary practice (51, 82, 114, 115). Exploiting the potential of web-based pet health information sources might be an appropriate first step to utilize the advantages of digital change for the pet owner-veterinarian relationship.

Recommending a set of reliable web-information sources and adopting empathic approaches seem to be appropriate ways of supporting RCC and shared decision-making. Conversely, the promotion and implementation of RCC in veterinary practice might open the actual and upcoming generations' minds toward the chances given by the web.

Although the results of this study could shed some light on the actual state of mind within the German veterinarian profession, a number of new questions were raised. First, it seems valuable to further identify the pet owner's need influencing their information collection. Why do they prefer to consult an information website or forum instead of asking their veterinarian for advice/discuss their questions or complaints? Which needs remain unfulfilled after a consultation that makes pet owners consult other information sources? Are there barriers that could be removed by improving our way of communication and/or our information-giving behavior? Additionally, we need to further evaluate the veterinarians' concrete concerns and barriers regarding the usage of web-based information sources.

The results of this study depict an incomplete perception of the advantages and risks that seem to accompany the phenomenon of self-educated pet owners. The findings imply that while veterinarians may view the potential benefits of the web as a valuable information source, there seem to be barriers that preventing it effective use. Since the potential risks cannot be denied, a focus on finding ways to break down those barriers seems urgent in the author's opinion. Research is needed to better understand the dynamics of web-based self-education and its impact on veterinarian work. Political efforts and targeted educational programs to implement changes in veterinary practice are also required. Improving the amount and accessibility of reliable and layperson-friendly veterinary medical web content appears necessary to avoid uncertainties and to reduce the long-lasting negative effects on veterinarian-pet owner relationships. Therefore, the political promotion of large-scale interdisciplinary cooperation of veterinarians, educational establishments, experts in web design, and online marketing seems to offer the most promising approach to successfully ensure a lasting improvement in the situation.

## Data Availability Statement

The raw data supporting the conclusions of this article will be made available by the authors, without undue reservation.

## Ethics Statement

Ethical review and approval was not required for the study on human participants in accordance with the local legislation and institutional requirements. The patients/participants provided their written informed consent to participate in this study.

## Author Contributions

AK conceived and designed the study, developed the theoretical framework, and implemented it into the preliminary model and questionnaire. Statistical preliminary considerations and coding of the SAS code were performed in close cooperation with RM. AK drafted and revised the manuscript. RM supervised and supported the project at each point of the development, conduction, statistical evaluation, and paper writing process. All authors contributed to the article and approved the submitted version.

## Conflict of Interest

AK was temporarily employed in a Start-up business with interest in Digital Animal Health Care (vetevo GmbH) that potentially could have been interested in the study results. The employment relationship started almost 1 year after the start of the research project and ended before publications were done. The company was not involved in any steps of study design, data collection or evaluation and no data or findings were provided to the company. Potential conflicts were prevented by obligation toward the privacy statements as well as the policies of good scientific work of the Institute for Veterinary Epidemiology and Biostatistics. The remaining author declares that the research was conducted in the absence of any commercial or financial relationships that could be constructed as a potential conflict of interest.
